# Depressive symptom mediates the association between the number of chronic diseases and cognitive impairment: a multi-center cross-sectional study based on community older adults

**DOI:** 10.3389/fpsyt.2024.1404229

**Published:** 2024-07-17

**Authors:** Li-Chong Lai, Dong-Mei Huang, Jie Peng, Xiao-Ying Cao, Xiao-Ling Feng, Pin-Yue Tao, Xiao Pan, Qi-Ni Pan, Deng-Jing Fan, Shu-Yu Lu, Cai-Li Li, Yan-Fei Pan, Peng-Xin Dong, Yi-Dan Chai, Ping- Huang, Hai-Chen Wu, Hui-Qiao Huang

**Affiliations:** ^1^ Nursing Department, The Second Affiliated Hospital of Guangxi Medical University, Nanning, Guangxi, China; ^2^ Rehabilitation Department, The Second Affiliated Hospital of Guangxi Medical University, Nanning, Guangxi, China; ^3^ Anesthesiology Department, The Second Affiliated Hospital of Guangxi Medical University, Nanning, Guangxi, China; ^4^ Ear, Nose, Throat, Head and Neck Surgery, The Second Affiliated Hospital of Guangxi Medical University, Nanning, Guangxi, China; ^5^ Party Committee Office, The Second Affiliated Hospital of Guangxi Medical University, Nanning, Guangxi, China

**Keywords:** multiple chronic conditions (MCC), depressive symptom, cognitive impairment, older adults, mediating effect

## Abstract

**Objective:**

The purpose of this study was to understand the relationship between the multiple chronic conditions (MCC), mental health and cognitive function of older adults in the community, and to propose a hypothesis that depressive symptom mediate the number of chronic diseases and cognitive impairment in older adults.

**Method:**

Participants aged 65 years and older from 35 communities in 14 cities in Guangxi, China were recruited. The residents’ depressive symptom (PHQ-9) and cognitive status (AD-8) were evaluated, Chi-square test was used to explore the effects of different socio-demographic characteristics on depressive symptom and cognitive impairment. Pearson correlation analysis and the process model 4 were used to explore the relationship between the number of chronic diseases, depressive symptom and cognitive impairment.

**Result:**

A total of 11,582 older adults were included in our analysis. The rate of MCC reaching 26.53%. Hypertension combined with diabetes accounts for the highest proportion of two chronic diseases (13.2%). Among the combination of three chronic diseases, the highest incidence of coexisting hypertension combined with cervical/lumbar spondylosis, and rheumatoid arthritis (7.1%). In this study, depression symptoms accounted for 12.9% of older adults aged 65 and above, and cognitive impairment accounted for 27.4%. Female, older age, reside in urban areas, lower educational levels, no spouse, live alone, and MCC were risk factors for depressive symptom and cognitive impairment in older adults (*P*<0.05). Depressive symptom had a mediating effect in the number of chronic diseases and cognitive impairment, and the mediating effect (1.109) accounted for 44.13% of the total effect (0.247).

**Conclusion:**

The mental health of the older adult needs to be taken seriously, and improving depressive symptom can reduce the occurrence of cognitive impairment in older patients with MCC to a certain extent.

## 1 Introduction

With the deepening of aging society, cognitive impairment has become a silent epidemic among the older adult. The fight against Alzheimer’s disease (AD) is considered a public health priority by the World Health Organization (WHO) (1). As of 2019, there are at least 50 million AD patients worldwide, and it is expected to reach 152 million by 2050, while China will reach 28.98 million (2). Mild cognitive impairment (MCI) is a cognitive stage between normal cognition and dementia, and is the primary sign of cognitive change. Its further development will significantly increase the risk of AD and other dementia, and gradually evolve into functional disorders and irreversible severe cognitive damage (3). Changes in cognitive function in the older adult will have a huge impact on their quality of life and family burden, and also pose an increasingly large challenge to healthcare systems worldwide (4, 5). It is urgent to screen the older adult for cognitive impairment in the community and design targeted early intervention programs.

One-third of AD cases worldwide can be attributed to potentially modifiable risk factors. There is Level 1 evidence that depressive symptom can significantly increase the risk of AD (6). And the conversion rate from MCI patients with depressive symptom to AD (31.0%) is significantly higher than that of MCI patients without depressive symptom (13.5%) (7). Long-term use of certain antidepressants such as anticholinergic drugs may also increase the risk of AD (8). In addition, the severity of cognitive impairment was positively correlated with the frequency of depression in a dose-response relationship (9). Studies have shown that older MCI patients are often accompanied by symptoms of depression, which is also an important reason for the decline in their quality of life, and it is necessary to pay attention to the synergistic intervention of the two (10).

Currently, medical services for the older adult are shifting from a disease-centered single-disease model to a patient-centered multi-disease model. How to improve the quality of life of older adult patients with MCC is a major issue in the aging society (11). Multiple chronic conditions (MCC) mean that one patient has two or more chronic diseases (12), and the prevalence rate of MCC among the older adult in China is 65.14% (13). Compared with a single chronic disease, patients with MCC have a rapid decline in physical function, a continuous decline in quality of life, including a 94% increased risk of functional limitation and a 73% increased risk of death (14). Research shows that MCC such as hypertension, diabetes, and chronic kidney disease have common etiological mechanisms such as vascular and metabolic factors, which increases the risk of cognitive impairment (15, 16), indicating that there may be synergy between various chronic diseases on the impact of cognitive function. It is particularly important to explore the common type of chronic diseases among older adults living in the community and further study the co-pathogenesis of MCC and cognitive impairment.

Existing research has mainly focused on exploring the cognitive function of specific chronic disease patients, while overlooking the impact of MCC on cognition. Although some scholars have demonstrated a close relationship between cognitive function and depressive symptom, the combined effect of MCC and depressive symptom on cognition has not been considered. The influence of older adult’s mental health on the relationship between the coexistence of multiple chronic diseases and cognitive impairment remains is unclear. Our study hypothesizes that depressive symptom may mediate the number of chronic diseases and cognitive dysfunction in the older adult. Based on the perspective of cognitive impairment in older adults with MCC, we seek to provide a reference for the development of targeted, multidisciplinary comprehensive prevention and management measures.

## 2 Materials and methods

### 2.1 Sample

This study is a cross-sectional study that recruited older adults at 35 community and village committee health centers in 14 cities in Guangxi, China, from March to October 2023. Health examinations and questionnaire surveys were conducted, and some objective information was provided by their primary caregivers. The eligible samples for inclusion were (1) 65 years old and older, (2) resident population living in the sampled community. And exclusion criteria were (1) the presence of conditions affecting the assessment, such as congenital or acquired developmental delays, corrected vision/hearing abnormalities, (2) did not complete all investigations and withdrew midway. All data were evaluated and collected by health professionals who have undergone unified training. A total of 12769 older adult’s health data were collected. All information is entered into the database through the electronic questionnaire system. During data cleaning, we excluded objects with missing data in three categories: chronic disease status, depressive symptom and cognitive function. Finally, a total of 11582 participants (90.7%) were included in this study for analysis. This study was approved by the Research Ethics Committee of the Second Affiliated Hospital of Guangxi Medical University (No. 2023-KY0905) and received informed consent from all participants.

### 2.2 Data measurement

#### 2.2.1 Measurement of chronic diseases

Data on chronic diseases were obtained through the following question: “Have you ever been informed by a doctor or other healthcare professional that you have the following chronic diseases?” Older adults and their caregivers answered yes or no. The list of chronic diseases was designed based on the 10th revision of the International Classification of Disease, 10th Revision (ICD-10). According to previous epidemiological research and prevalence rates (17), we selected the following 20 chronic diseases and symptoms: hypertension, heart disease/coronary heart disease, diabetes, cerebrovascular disease, chronic bronchitis, cancer, kidney disease, liver disease, gastroenteritis or other digestive diseases, tuberculosis, rheumatoid arthritis, cervical/lumbar spondylosis, reproductive system diseases, prostate disease, urinary system diseases, glaucoma or cataracts, osteoporosis, emotional and mental problems, neurological diseases, and hearing loss. If any one of these diseases was diagnosed by a hospital, the participant was defined as having a chronic disease. Those with two or more chronic diseases were considered as having MCC, while those with less than 2 chronic diseases were considered as non-MCC patients (18).

#### 2.2.2 Measurement of depressive symptom

The Patient Health Questionnaire-9 (PHQ-9) is an effective scale for assessing depressive symptom based on the Diagnostic and Statistical Manual of Mental Disorders (DSM)-V. It measures participants’ depressive symptom over the past two weeks and scores 9 items from 0 (not at all) to 3 (nearly every day), with a total score of 27. A score of ≥5 indicates the presence of depressive symptom (19).

#### 2.2.3 Measurement of mild cognitive impairment

We use the Ascertain dementia-8 (AD-8) to measure cognitive function in the older adult and invited their primary caregivers to fill out this questionnaire. The AD-8 is a simple and rapid screening tool that is sensitive to early cognitive changes in many common dementia diseases (20). The AD-8 includes eight items that evaluate the judgment, daily living ability, initiative, orientation, and memory, and each item is scored as 0 or 1. The total score of the AD-8 ranges from 0 to 8, with higher scores indicating greater cognitive impairment. A score of ≥2 indicates the possible presence of cognitive impairment (21).

### 2.3 Statistical analysis

All analyses were conducted using SPSS 23. Measurement data is expressed as mean and standard deviation, while count data is expressed as frequency and percentage. The Chi-square test was used to explore the effects of different socio-demographic characteristics on depressive symptom and cognitive impairment. Pearson correlation coefficient was used to conduct bivariate correlation analysis, and Model 4 in the Process program was used to examine the mediating role of depressive symptom between the number of chronic diseases and cognitive function in the older adult. Significance tests were two-tailed with a significance level of 0.05. Bootstrapping was used for significance testing for the mediation analysis.

## 3 Results

### 3.1 Characteristics of study population

A total of 11582 participants were included in analysis with the age of 65 and over. Among them, there are 5118 males (44.2%) and 6464 females (55.8%). The mean age was (73.74 ± 6.87) years old. There are 6223 (53.7%) residing in urban areas and 5359 (46.3%) residing in rural areas. The average length of schooling was (5.19 ± 3.92) years. More than half of participants have a spouse (68.7%) and a small percentage (8.4%) are living alone.

### 3.2 Common type of MCC in the older adults

Among the 20 chronic diseases surveyed, the top three with the highest prevalence were hypertension (37.6%), cervical/lumbar spondylosis (15.3%), and rheumatoid arthritis/joint disease (11.0%), while the remaining 17 were all below 10% (**Table 1**). The incidence rate of MCC was 26.53%, with the number of older adults suffering from 0, 1, 2, 3, and more than 4 chronic diseases were 3677 (31.7%), 4332 (37.4%), 2261 (19.5%), 898 (7.8%), and 414 (3.6%), respectively. Among the combinations of chronic diseases, there were 138 types of two chronic diseases, the three combinations with the highest proportion were hypertension with diabetes (13.2%), hypertension with cervical/lumbar spondylosis (9.9%), and hypertension with cerebrovascular disease (8.4%). It’s worth noting that the top five all contain hypertension. There were 192 types of three chronic diseases, with the highest prevalence combination were hypertension combined with cervical/lumbar spondylosis and rheumatoid arthritis/joint disease (7.1%), hypertension combined with diabetes and heart disease (6.2%) (**Table 2**).

**Table 1:** The prevalence of 20 chronic diseases among the older adult.

sort	Chronic disease	Number	Proportion(%)
1	Hypertension	4352	37.6
2	Cervical/lumbar spondylosis	1773	15.3
3	Rheumatoid/arthritis	1272	11.0
4	Diabetes	948	8.2
5	Gastroenteritis	814	7.0
6	Osteoporosis	777	6.7
7	Heart disease	748	6.5
8	Cerebrovascular diseases	634	5.5
9	Chronic bronchitis	443	3.8
10	Glaucoma or cataracts	432	3.7
11	Deaf	385	3.3
12	Neurological disorders	205	1.8
13	Cancer/Tumor	130	1.1
14	Kidney disease	103	0.9
15	Liver disease	75	0.7
16	Prostatic diseases	71	0.6
17	Urinary system diseases	56	0.5
18	Tuberculosis	37	0.3
19	Emotional and mental issues	34	0.3
20	Reproductive system diseases	11	0.1

**Table 2:** Top 5 Combination Patterns of 2 MCC and 3 MCC n (%).

sort	MCC of two chronic diseases N=2261		MCC of three chronic diseases N=898	
	type of MCC	Number(%)	type of MCC	Number(%)
1	Hypertension+Diabetes	298(13.2%)	Hypertension+Cervical/lumbar spondylosis+Rheumatoid/arthritis	64(7.1%)
2	Hypertension+Cervical/lumbar spondylosis	224(9.9%)	Hypertension+Diabetes+Heart disease	56(6.2%)
3	Hypertension+Cerebrovascular disease	190(8.4%)	Hypertension+Rheumatoid/arthritis+Osteoporosis	54(6.0%)
4	Hypertension+Heart disease	179(7.9%)	Chronic bronchitis +Gastroenteritis+ Rheumatoid/arthritis	35(3.9%)
5	Hypertension+Rheumatoid/arthritis	155(6.9%)	Hypertension+Diabetes+Cerebrovascular disease	33(3.7%)

### 3.3 The prevalence and influencing factors of depressive symptom and cognitive impairment in older adults

In this study, depressive symptom accounted for 12.9% of older adults aged 65 and above, and cognitive impairment accounted for 27.4%. In the univariate analysis, female, older age, reside in urban areas, lower educational levels, no spouse, live alone, and MCC were risk factors for depressive symptom and cognitive impairment, and the difference is statistically significant (*p* < 0.05) (**Table 3**).

**Table 3:** The univariate analysis of depressive symptom and cognitive impairment in the older adult.

Variables	depressive symptom [n(%)]			*χ^2^*	*P*	Cognitive function[n(%)]		*χ^2^*	*P*
	Total 11582	Normal 10089(87.1)	Depression 1475(12.9)			Normal 8407(72.6)	Cognitive impairment 3175(27.4)		
Gender									
Male	5118	4563(89.2)	555(10.8)	35.291	<0.001	3992(78.0)	1126(22.0)	135.006	<0.001
Female	6464	5522(85.4)	942(14.6)			4415(68.3)	2049(31.7)		
Age									
65-70	4665	4251(91.1)	414(8.9)	241.296	<0.001	3758(80.6)	907(19.4)	443.762	<0.001
71-79	4556	3992(85.9)	564(14.1)			3301(72.5)	1255(27.5)		
≥80	2361	1842(71.8)	519(28.2)			1348(57.1)	1013(42.9)		
Residence									
City	6223	5295(85.1)	928(14.9)	47.19	<0.001	4460(71.7)	1763(28.3)	5.686	0.017
Rural	5359	4790(89.4)	569(10.6)			3947(73.7)	1412(26.3)		
Educational period									
0	2175	1710(78.6)	465(21.4)	246.359	<0.001	1240(57.0)	934(43.0)	443.528	<0.001
1-6	5766	4995(86.6)	771(13.4)			4165(72.2)	1601(27.8)		
≥7	3641	3380(92.8)	261(7.2)			3002(82.4)	639(17.6)		
marital status									
married	7985	7152(89.6)	833(10.4)	142.001	<0.001	6069(76.0)	1916(24.0)	150.975	<0.001
No spouse	3597	2933(81.5)	664(18.5)			2338(65.0)	1259(35.0)		
Living conditions									
Live alone	995	843(84.7)	152(15.3)	5.346	0.021	674(67.7)	321(32.3)	12.857	<0.001
Live together	10587	9242(87.3)	1345(12.7)			7733(73.0)	2854(27.0)		
Number of chronic diseases									
0	3677	3538(96.2)	139(3.8)	546.250	<0.001	3015(82.0)	662(18.0)	414.345	<0.001
1	4332	3712(85.7)	620(14.3)			3152(72.8)	1180(27.2)		
2	2261	1843(81.5)	418(18.5)			1479(65.4)	782(34.6)		
3	898	719(80.0)	179(20.0)			575(64.0)	323(36.0)		
≥4	414	273(65.9)	141(34.1)			186(44.9)	228(55.1)		
Whether MMC									
MMC	3073	2835(76.0)	738(24.0)	274.303	<0.001	2240(72.9)	1333(27.1)	254.212	<0.001
Non-MCC	8009	7250(90.5)	759(9.5)			6167(77.0)	1842(23.0)		
Type of MMC									
Hypertension+diabetes	298	262(88.8)	33(11.2)	32.255	<0.001	218(94.3)	77(5.7)	17.769	0.001
Hypertension+cervical/lumbar spondylosis	224	183(81.7)	41(18.3)			156(69.6)	68(30.4)		
Hypertension+cerebrovascular disease	190	133(70)	57(30)			111(58.4)	79(41.6)		
Hypertension+heart disease	179	155(86.6)	24(13.4)			126(70.4)	53(29.6)		
Hypertension+rheumatoid/arthritis	155	120(77.4)	35(22.6)			93(60.0)	62(40.0)		

### 3.4 Correlation of the number of chronic diseases, depressive symptom and cognitive impairment in the older adult

**Figure 1:**
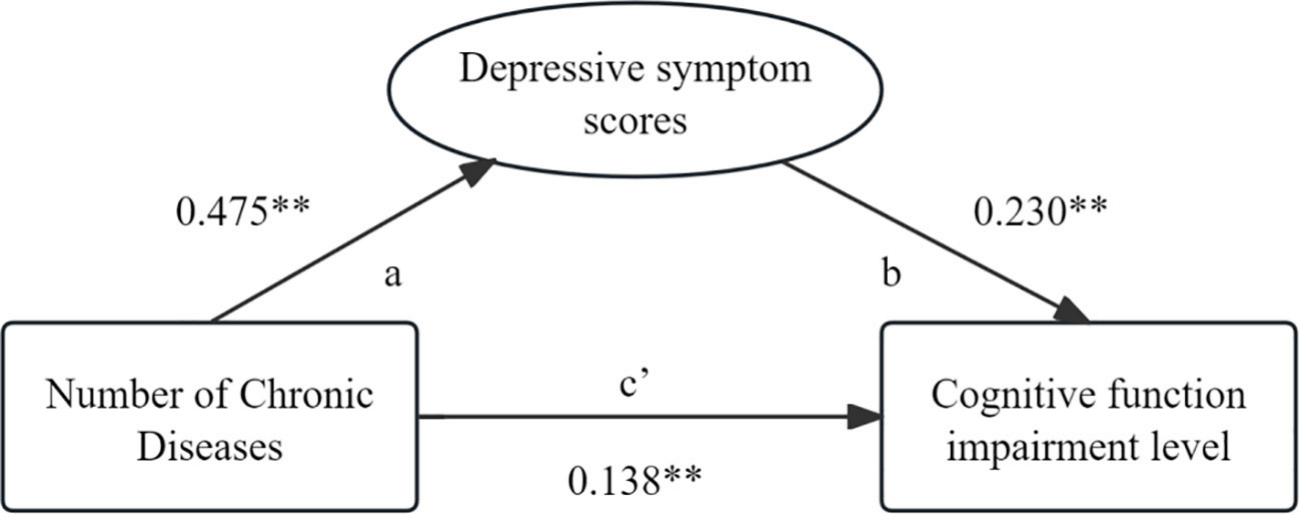
Mediation model path diagram. **p < 0.001.

The statistical analysis result shows that the number of chronic diseases is positively correlated with depressive symptom and cognitive function (*p*<0.05), and depressive symptom are also positively correlated with cognitive impairment (*p*<0.05) **Table 4**. After controlling for six variables that have an impact on cognitive impairment, including gender, age, place of residence, the education level, marital status, and whether living alone, a three-step test was conducted to examine the mediating effect of depressive symptom between the number of chronic diseases and cognitive impairment. In Model 1 test, the number of chronic diseases had a significant predictive effect on cognitive impairment(β=0.247, *p*<0.001) indicates that the total effect is valid. In the test of Model 2, the number of chronic diseases has a significant impact on depressive symptom(β=0.475, *p*<0.001) indicates that path a is valid. Finally, in the Model 3 test, after controlling for the influence of the mediating variable (depressive symptom), the number of chronic diseases had a significant direct effect on cognitive function (β=0.138, *p*<0.001), indicating that path c’ was effective. After controlling for the effect of the number of chronic diseases, the mediating variable (depressive symptom) had a significant effect on cognitive impairment (β=0.230, *p*<0.001), suggesting that path b was effective (**Table 5**). In conclusion, the mediating effect of depressive symptom between the number of chronic diseases and the impairment of cognitive function are established, and the mediating effect (0.109) accounted for 44.13% of the total effect (0.247) (**Table 6**; **Figure 1**).

**Table 4:** The correlation between number of chronic diseases, depressive symptom and cognitive impairment.

Variables	M	SD	Depressive symptom	Cognitive impairment	Number of Chronic Diseases
Depressive symptom	1.22	2.301	1.000		
Cognitive impairment	1.20	1.907	0.361**	1.000	
Number of Chronic Diseases	1.159	1.129	0.237**	0.191**	1.000

**p < 0.001. M, mean value; SD, standard deviation.

**Table 5:** Process distribution return.

step	Cause variable	Independent variable	R	R-sq	F	β	t
Model1	Cognitive impairment	Number of Chronic Diseases	0.317	0.100	165.099	0.247	17.173
Model2	Depressive symptom	Number of Chronic Diseases	0.275	0.076	120.807	0.475	24.252
Model3	Cognitive impairment	Number of Chronic Diseases	0.434	0.189	300.941	0.138	9.810
		Depressive symptom				0.230	33.561

**Table 6:** Bootstarp intermediary effect test results.

Effect	effect value	SE	LLCI	ULCI	Effect proportion
Direct effect	0.138	0.013	0.110	0.165	55.78%
Indirect effect	0.109	0.005	0.095	0.124	44.13%
Total effect	0.247	0.014	0.218	0.275	

SE, the standard error of indirect effects estimated; LLCI, lower limit confidence interval; ULCI, upper limit confidence interval.

Exposure: Number of Chronic Diseases; outcome: Cognitive impairment; Adjusted for gender, age, place of residence, the education level, marital status, and whether living alone.

## 4 Discussion

In this survey, in addition to the most commonly occurring hypertension, musculoskeletal diseases (neck/lumbar spine disease, rheumatoid arthritis/arthritis, osteoporosis) were reported the most. These diseases are closely related to physical activity and can cause significant difficulties in the daily life of the older adults (22). In future research, more attention should be paid to chronic damage to the skeletal muscles of the older adult and the development of exercise rehabilitation plans to alleviate pain. In the type of MCC, older adults with hypertension combined with cerebrovascular disease have the highest proportion of depressive symptom and cognitive impairment, followed by hypertension combined with rheumatoid arthritis. The reason may be that patients with cerebrovascular diseases and musculoskeletal diseases have to constantly endure the pain caused by the disease in their daily activities. If it worsens, they also face a high risk of disability. The accumulation of stress and emotions can worsen the patient’s mental health (23). The changes in cerebral blood vessels and inflammatory factors are both potential mechanisms for cognitive impairment (24). Meanwhile, the ranking of depressive symptom and cognitive impairment in each type of MCC is basically the same, which lays a foundation for the hypothesis made in this study.

There is a significant correlation between the number of chronic diseases, depressive symptom, and cognitive impairment in the older adult, and depressive symptom partially mediates the relationship between the number of chronic diseases and cognitive impairment. The coexistence of multiple chronic diseases can accelerate the functional decline of various systems in the body, leading to a sustained state of chronic consumption, which reduces the body’s resistance and tolerance to the external environment (25, 26). Moreover, the long-term evolution of diseases can cause damage to the body’s vessels and nerves, leading to a decline in cognitive function (27). The results of this study showed that the higher number of chronic diseases, the higher incidence of depression, which is consistent with a longitudinal study of older adults in China, and shows a dose-response relationship (28). Studies have shown that the potential pathogenesis of cognitive decline, such as non-physiological in aging, cerebrovascular disease, and leukoaraiosis, is related to the pathogenesis of depression (29–31). Ruan (32)suggested that depression can cause the body to produce various chronic inflammatory factors, which through the blood-brain barrier cause an increase in amyloid precursor proteins in the brain, leading to a decline in cognitive function. And there is data to suggest that depression still exists on the devastating role of on basic cognitive capacities in older adults without other comorbid disorders (33). To sum up, the hypothesis of this study is valid and can be explained. Due to the survey targeting older adults in the community, comorbidity index was not calculated, and the impact of comorbidity severity on depressive symptom could not be determined. In summary, older adults with MCC are plagued by various diseases and complications for a long time. Persistent physical symptoms combined with reduced social participation have a negative impact on their mental health (34). Adverse emotions such as depression can reduce the subjective memory and cognitive flexibility of the older adult, leading to cognitive changes (35).

Therefore, older adults healthcare workers should not only treat the disease itself, but also pay more attention to the psychological state of patients to reduce the occurrence of cognitive impairment. The factors related to depressive symptom in this study showed that female, advanced age, low education, and no spouse were risk factors. Similar to previous research results (36, 37), it suggests that we should fully consider the impact of different age groups on psychological distress (38) and take proactive and effective measures for precise intervention. Targeted health education should be provided during free clinics or physical examinations for the older adult to improve their health literacy and compensate for the psychological impact of low education (39). Encourage older adults to actively participate in social activities and help them build a positive social support network. In this study, the detection of depressive symptom in older adults living in urban areas was higher than in rural areas, which is inconsistent with previous studies (40). The reason may be that rural older adults have been in a relatively difficult environment for a long time, are more easily satisfied with themselves and the environment, have strong independence and autonomy, which may enhance their psychological resilience and adaptability, thereby reducing psychological distress (41).

Finally, this study has certain limitations. As is well known, cognitive symptoms are associated with some clinical variables, such as the disease course, acute onset, or remission period, at the same time the proportion of chronic disease types may also have an impact on the outcome of the mediating effect, but unfortunately, we did not consider these factors. Therefore, research should be conducted on the impact of the severity of MCC on depressive symptom and cognitive impairment to further confirm these findings. Secondly, this study adopts a cross-sectional design and only analyzes the mediating effect of the occurrence of cognitive impairment in the older adult, without elaborating the causal relationship. Finally, because this study was conducted in the community through interviews, older adult’s memories of diseases may not be comprehensive enough, resulting in recall bias. And the assessment of depression and cognitive impairment may be subjective by investigators, which may affect the accuracy of the data. In the future, further related intermediary studies can be conducted in hospitals on older adults with MCC.

## 5 Conclusions

It is challenging for older adults with MCC to implement cognitive interventions considering multiple chronic diseases at the same time. It is necessary to identify modifiable intermediate variables for secondary prevention in order to reduce or delay the onset of cognitive impairment. Our study found that depressive symptom has a mediating effect between the number of chronic diseases and cognitive impairment. Therefore, focusing on the mental health of older adults can reduce the occurrence of cognitive impairment in MCC old adults. In the process of policy implementation, cognitive impairment screening should be coordinated with chronic disease management, emphasizing the prevention and intervention of cognitive related diseases, and promoting the development of healthy aging.

## Data availability statement

The raw data supporting the conclusions of this article will be made available by the authors, without undue reservation.

## Ethics statement

The studies involving humans were approved by the Second Affiliated Hospital of Guangxi Medical University Ethical Review Committee. The studies were conducted in accordance with the local legislation and institutional requirements. The participants provided their written informed consent to participate in this study. The manuscript presents research on animals that do not require ethical approval for their study.

## Author contributions

L-CL: Writing – original draft, Investigation. DMH: Data curation, Writing – original draft. JP: Investigation, Writing – original draft. X-YC: Investigation, Writing – original draft. X-LF: Investigation, Writing – original draft. P-YT: Formal analysis, Writing – review & editing. XP: Formal analysis, Writing – review & editing. Q-NP: Methodology, Writing – review & editing. D-JF: Methodology, Writing – review & editing. S-YL: Methodology, Writing – original draft. C-LL: Writing – review & editing. Y-FP: Writing – review & editing. P-XD: Investigation, Writing – original draft. Y-DC: Investigation, Writing – original draft. P-H: Investigation, Writing – original draft. H-CW: Investigation, Writing – original draft. H-QH: Project administration, Supervision, Writing – review & editing.
